# Descriptive Catalogue of the Preparations in the Museum of the Royal College of Surgeons in Ireland

**Published:** 1841-04

**Authors:** 


Art. IX.—*Descriptive Catalogue of the Preparations in the Museum of the Royal College of Surgeons in Ireland.* By John Houston, m.d. m.r.i.a., Curator of the Museum. Vol. II. (Pathology.)—*Dublin,* 1840. 8vo, pp. 604.

The publication of this work is extremely honorable to the Dublin College of Surgeons, and the manner of its execution reflects the highest credit on Dr. Houston. The following extracts from the preface give an account of the plan of the work, (which is excellent,) and supersede the necessity of our entering into any details respecting its contents. We regret, however, that want of room prevents us from enriching our pages with a few of the interesting and important histories which abound in it. We will only further add that the "Descriptive Catalogue" ought to occupy a place in every medical library, and deserves to be consulted by every pathologist.

"The book is divided into two essential parts—the index which is placed at the end, and the descriptive part. The index contains a brief list of all the preparations in the museum, and demonstrates as nearly as possible the order of their arrangement on the shelves; it also indicates the page in the descriptive division of the work, at which a further notice of particular preparations may be found.

"The preparations are distributed into six classes, which are severally distinguished, both in the catalogue and in the presses, by letters of the alphabet in Roman characters. The first class, distinguished by the letter A, contains all the organs concerned in the assimilation of food The second, by B, contains the organs of circulation. The third, by C, the organs of circulation. The fourth, by D, the organs of sense. The fifth, by E, the organs of locomotion and prehension. The sixth, by F, the organs of generation and secretion of urine. These classes are again subdivided into orders, the numbers of which vary with the most convenient subdivisions which each admits of, and are distinguished by letters in italics. Thus, the first class, embracing all the series of organs concerned in the assimilation of food, and distinguished by the Roman letter A, admits conveniently of subdivision into four orders. In the first, marked *a*, are contained those parts of the alimentary system which, in the form of preparations, can be most conveniently grouped and exhibited together, viz. the mouth, tongue, pharynx, and œsophagus. The second order, *b*, contains preparations of the stomach. The third, *c*, contains those of the intestines. The fourth, *d*, the glands connected with the intestines. The same mode of subdivision into orders, and of marking by appropriate letters in italics, is observed throughout the other classes.

"Every exertion has been made to secure an accurate report both of the history and recent pathological appearances of every preparation. The account given of each is, in fact, a condensed abstract, made by the author from the most authentic sources, viz.—either from the verbal or written communication of the donor, or from some printed statement authorized by him,—all such abstracts being studiously curtailed to such dimensions, that, while connecting intelligibly the post-mortem appearances with the history of the disease during life, they might not be unsuited to the pages of a work like the present."

